# Compact UWB MIMO Antenna for 5G Millimeter-Wave Applications

**DOI:** 10.3390/s23052702

**Published:** 2023-03-01

**Authors:** Mohamed Atef Abbas, Abdelmegid Allam, Abdelhamid Gaafar, Hadia M. Elhennawy, Mohamed Fathy Abo Sree

**Affiliations:** 1 Department of Electronics and Communications Engineering, Ain Shams University, Cairo 11566, Egypt; 2 Department of Electronics and Communications Engineering, Arab Academy for Science, Technology and Maritime Transport, Cairo 11799, Egypt; 3 Head of Information Technology, GUC, Cairo 101516, Egypt

**Keywords:** MIMO antenna, UWB, 5G, DGS, ECC, CCL, FDTD

## Abstract

This paper presents a printed multiple-input multiple-output (MIMO) antenna with the advantages of compact size, good MIMO diversity performance and simple geometry for fifth-generation (5G) millimeter-wave (mm-Wave) applications. The antenna offers a novel Ultra-Wide Band (UWB) operation from 25 to 50 GHz, using a Defective Ground Structure (DGS) technology. Firstly, its compact size makes it suitable for integrating different telecommunication devices for various applications, with a prototype fabricated having a total size of 33 mm × 33 mm × 0.233 mm. Second, the mutual coupling between the individual elements severely impacts the diversity properties of the MIMO antenna system. An effective technique of orthogonally positioning the antenna elements to each other increased their isolation; thus, the MIMO system provides the best diversity performance. The performance of the proposed MIMO antenna was investigated in terms of S-parameters and MIMO diversity parameters to ensure its suitability for future 5G mm-Wave applications. Finally, the proposed work was verified by measurements and exhibited a good match between simulated and measured results. It achieves UWB, high isolation, low mutual coupling, and good MIMO diversity performance, making it a good candidate and seamlessly housed in 5G mm-Wave applications.

## 1. Introduction

There is no doubt that this era is one of wireless communication technology that provides its contributed to the development of very high data rates, higher capacity, and reliability in communication applications used daily by a wide range of people [1,2]. Recently, Ultra-Wide Band (UWB) technology has been addressed in wireless communication systems due to its high data rates and low fabrication cost. Unfortunately, reflection and diffraction of an electromagnetic wave are the main obstacles in the dense medium, which cause multipath fading problems in UWB technology. Multiple-Input Multiple-Output (MIMO) technology has been proposed to alleviate multipath fading and increase transmission quality in wireless communication systems [3,4]. MIMO antenna systems associated with UWB technology provide an increase in the capacity linearly with the number of antennas without the need to add an additional frequency spectrum or increase the power resources. However, the design is affected by the mutual coupling between the antenna elements and the spatial correlation between them [5,6].

Concerning the separation between the antenna elements, the mutual coupling is inversely proportional to it. However, this distance should be chosen to be at a minimum between λ/4 and λ/2 to have a mutual coupling of less than −15 dB and therefore have an efficient working MIMO system. Additionally, a larger MIMO system size results from the increased separation between antenna units. As a result, while designing MIMO antennas, trade-offs between the total size of MIMO systems and the minimizing of mutual coupling should be taken into account [7]. 

Many researchers aim to enhance the performance of different parameters such as bandwidth, compact size, gain, efficiency, mutual coupling and diversity properties in the Fifth Generation (5G) Millimeter-Wave (mm-Wave) antenna design. So, they use several enhancement techniques to obtain the best performance, like substrate choice, corrugation, multi-element, dielectric lens and mutual coupling reduction techniques [8,9]. First and foremost, the substrate used in antenna fabrication is critical. For antenna fabrication, various substrates with varying permittivity and loss tangents are available. As a result, a substrate with a lower relative permittivity and a lower loss tangent will increase gain while decreasing power loss [10]. Second, the corrugation structure, which removes the metal part of the radiator’s edge, can help improve the bandwidth and front-to-back ratio [11]. In terms of the multi-element technique, it increases an antenna’s gain while also increasing its bandwidth and efficiency. As a result, structures can meet requirements such as high gain and wide bandwidth that a single antenna cannot. Furthermore, the dielectric lens can transmit electrostatic radiation in only one direction, increasing an antenna’s directivity and gain [12,13]. Dielectric lenses of various shapes are created using the same or different substrate material. Finally, mutual coupling reduction techniques mitigate the effect of multiple elements on antenna performance. This technique, also known as the isolation technique, is critical for achieving the best diversity performance from MIMO antennas.

A variety of approaches have been used to reduce mutual coupling between MIMO antenna elements. Neutralization techniques, simultaneous matching of orthogonal feeding or elements, pattern diversity, and significant polarisation are all included [11,12,13]. In addition, many methods have been employed in an attempt to reduce coupling, including Defective Ground Structures (DGS), Electromagnetic Band Gap (EBG) substrates, complementary split ring resonators (CSRR), metamaterials, and parasitic resonators [14,15,16,17]. These methods, however, call for a sizable amount of circuit board space. Etching the isolating slots between the antenna elements is a different strategy. Numerous slot configurations, such as an F-shaped stub, a T-shaped slot, an L-shaped slot, or a vertical slot, have been tested [18,19,20,21]. Although the mutual coupling between the radiating antennas is reduced by these slots, the overall gain remains unchanged. In a 4-element MIMO antenna, linear slots were inserted in between the radiating slots to lessen mutual coupling [22]. In [23], etching linear slots between the radiators of a 4-element MIMO antenna system reduced the mutual coupling by an amount of 20 dB. A 4-element MIMO antenna with four T-shaped radiating elements was proposed by M. Alibakhshikenari [24]. This small, wideband antenna had a maximum gain of 5 dBi and a minimum isolation of 15 dB. This resulted from stubs on the bottom ground and a shortened ground plane. It was suggested in [25] to use a 2-port textile MIMO antenna with two half-ring-shaped antenna elements. In order to obtain a minimum isolation of 15.5 dB, the ground plane is defective. Dual patched wideband MIMO antennas with an inverted pair of L-shaped stubs in the ground were another design [26]. In comparison to other proposed designs, the isolation was roughly 20 dB, and the highest gain was 5.32 dBi. The main drawbacks of this system are that it is more complex and has low gain.

On the other hand, Diversity properties of MIMO systems like envelope correlation coefficient (ECC), Channel capacity loss (CCL), Diversity Gain (DG), Multiplexing Efficiency (ME) and Total Active Reflection Coefficient (TARC) are strongly affected by the mutual coupling between antenna elements of MIMO system [27,28,29,30]. Therefore, it is evident that a MIMO antenna system should have more diversity parameters to be evaluated in addition to the antenna’s parameters. These metrics are not required for single-antenna elements but are required for multi-antenna devices.

Although significant work and research were made in the last half-decade to introduce reliable MIMO antennas with acceptable diversity parameters, In this work, the DGS and antenna element orientation are developed to achieve acceptable isolation along the operating band. In this study, a 4-port MIMO antenna system covering the frequency range of 25 to 50 GHz is developed, constructed, and tested. The ground plane’s carved slots between the antenna elements and the orthogonal orientation of the antenna elements form the design’s foundation. A DGS is then used to deploy for mutual coupling reduction and gain enhancement after a reference antenna is tuned for bandwidth enhancement. The work is organised as follows; Section 2 presents the antenna design and optimization of a single antenna. Section 3, which is concerned with simulation and measurable outcomes, presents a brief summary of the theoretical basis, including diversity parameters. Section 5 serves as the paper’s conclusion.

## 2. Antenna Design

Since the antenna is the pivot element of MIMO antenna systems, many researchers present different antennas adapted for this technology. Concerning mm-Wave applications, printed antennas are the best candidates for this technology because of their low cost, low profile, compact size, and good choice to achieve a functional element with a proper balance of performance and manufacturing complexity for millimeter wave applications.

**Figure 1:**
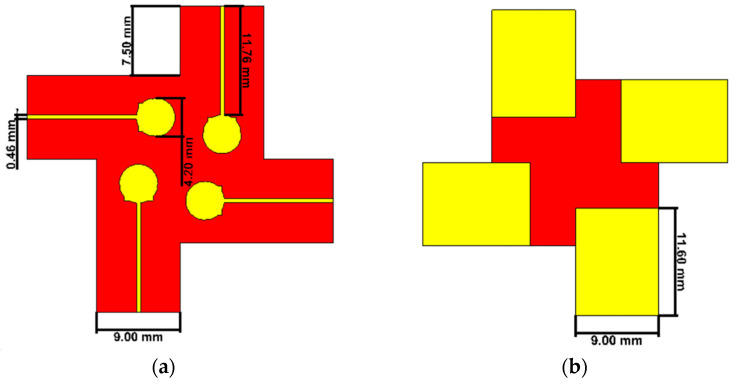
The proposed antenna (**a**) Top view. (**b**) Bottom view.

**Figure 2:**
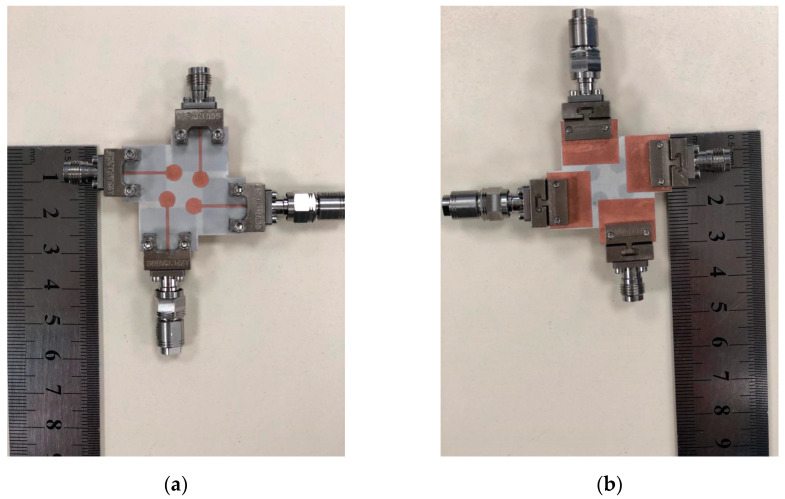
The fabricated prototype of 4 elements MIMO antenna (**a**) Top View (**b**) Bottom view.

**Figure 3:**
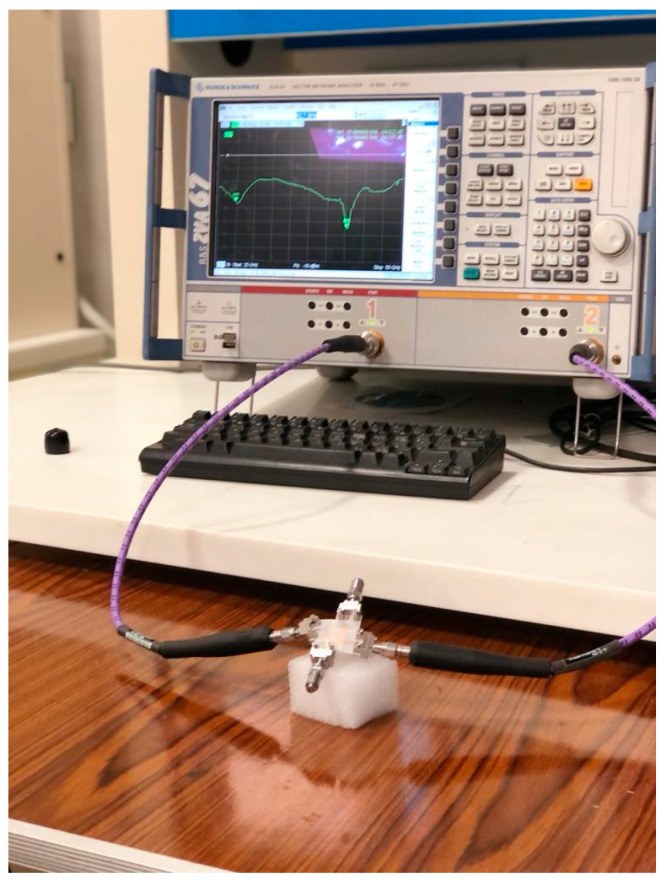
Measurement setup of proposed MIMO using ROHDE & SCHWARZ ZVA67 Vector Network Analyzer.

Figure 1 depicts the suggested four-element MIMO antenna design taken into consideration in this study. In (a), the top view is shown, while (b) shows the bottom perspective (b). The constructed model of the antenna structure is shown in Figure 2. The modelling and simulation of this design are done using the Finite-Difference Time-Domain (FDTD) (CST Microwave Studio). The choice of the substrate material, RO4003C, for electronic circuit boards that provide high-frequency performance and low-cost circuit manufacturing was made since it is one of the most widely used industry-wide standard substrate material formats [31,32]. Figure 3 illustrates the measurement setup of the constructed antenna using the ROHDE & SCHWARZ ZVA67 Vector Network Analyzer [33]. The dimensions of the MIMO are 33 × 33 × 0.203 ${\mathrm{mm}}^{3}$.

**Figure 4:**
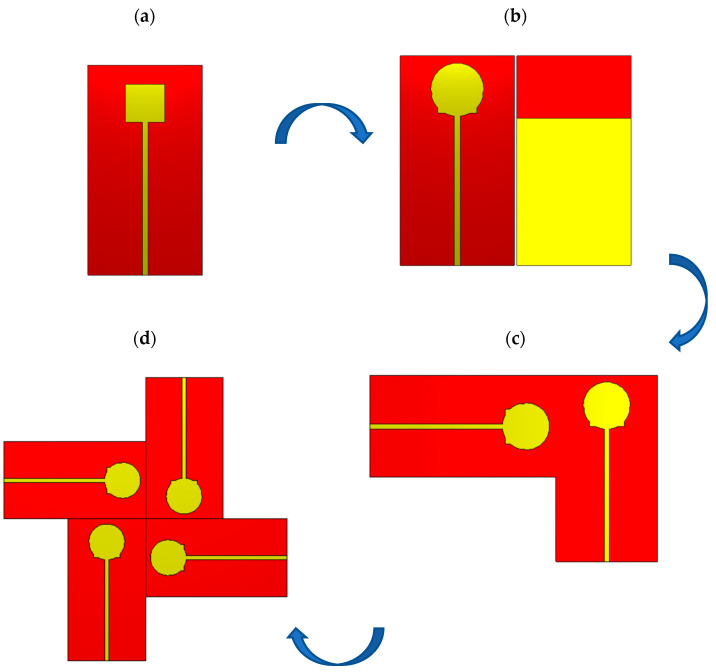
Steps of design (**a**) rectangular patch antenna with inset feed and full ground (**b**) single proposed antenna (top and bottom) (**c**) 2-port MIMO antenna with orthogonal orientation (**d**) Final step for 4-port MIMO antenna.

An overview of the sequential design evolution is shown in Figure 4. Figure 4 shows how the modelling begins with a single antenna element (a). as an initial step. Using well-known mathematical formulas, a rectangular patch antenna with inset feed and the full ground plane is produced it resonates at 30 GHz. The preliminary values of length L and width W can be determined from [34]:

(1) $$W=\frac{c}{2f_{r}\;}\sqrt{\frac{2}{\varepsilon_{r}+1}}$$

(2) $$L=L_{eff}-2\Delta L$$

(3) $$\Delta L=0.412h\frac{\left(\varepsilon_{reff}+0.3\right)\left(\frac{W}{h}+0.264\right)}{\left(\varepsilon_{reff}-0.258\right)\left(\frac{w}{h}+0.8\right)}$$

(4) $$\varepsilon_{reff}=\frac{\varepsilon_{r}+1}{2}+\frac{\varepsilon_{r}-1}{2}{\left(1+12\frac{h}{W}\right)}^{-0.5},\frac{W}{h}>1$$

(5) $$L_{eff}=\frac{c}{2f_{r}\sqrt{\varepsilon_{reff}}\;}$$

The rectangular patch antenna is improved for a larger band in the second step by etching the ground (DGS) and adding vertically and horizontally oriented circular slots to the three sides, as shown in Figure 4b. To reduce the mutual coupling, the MIMO structure is created for two ports utilizing an orthogonal orientation, as shown in Figure 4c. Based on this setup, Figure 4d shows a MIMO antenna structure with four ports to span the frequency range of 25 to 50 GHz.

**Figure 5:**
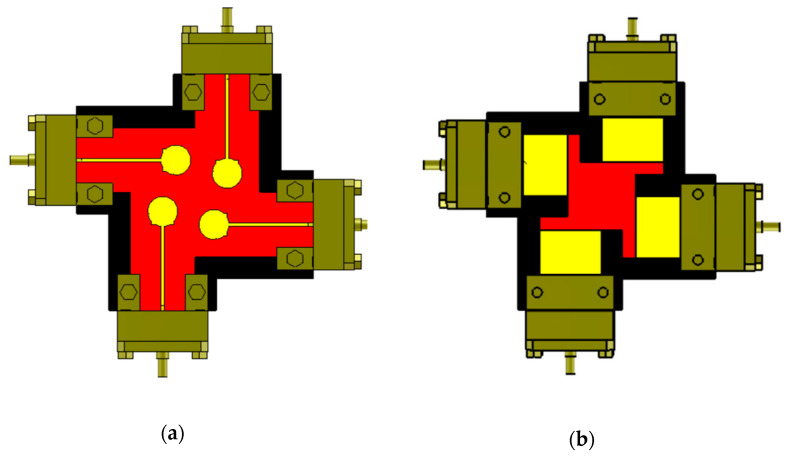
The prototype of 4 elements MIMO antenna with connector: (**a**) Top View (**b**) Bottom view.

The long microstrip feed in Figure 4b is a typical choice for measurements in the millimetric band. To accommodate the special connector needed for the antenna element, additional length and width would need to be added to the grounded substrate. Figure 5 demonstrates the fixation of the connector of each individual antenna element. In addition, a separation between the fixation nuts of the connector and the radiator of MIMO antenna should attain a long length to separate them as shaded by black color.

The fabrication process includes many steps. Based on the available dielectric substrate, we select the appropriate one suitable for the required frequency band. The antenna is modelled on CST microwave studio simulator. The optimization process is developed. Then, the Gerber file and “.Sab” file are exported to the manufacturer. At this step the duty of the academic researchers is finished. On the fabrication side the mask of design is developed. The copper is etched from the top and bottom surfaces using chemical process.

## 3. MIMO Antenna Parameters for Performance Evaluations

When presenting a MIMO antenna along with other fundamental characteristics, Diversity parameters analysis is a must in addition to the fundamental antenna performance evaluations and parameters, such as bandwidth, resonance frequency, radiation patterns, gain, and efficiency. These characteristics are necessary for multi-antenna devices but not for single-antenna elements. The parameters used for this include the TARC, CCL, DG, and ECC The next subsections go over these variables in relation to the suggested antenna design.

**Figure 6:**
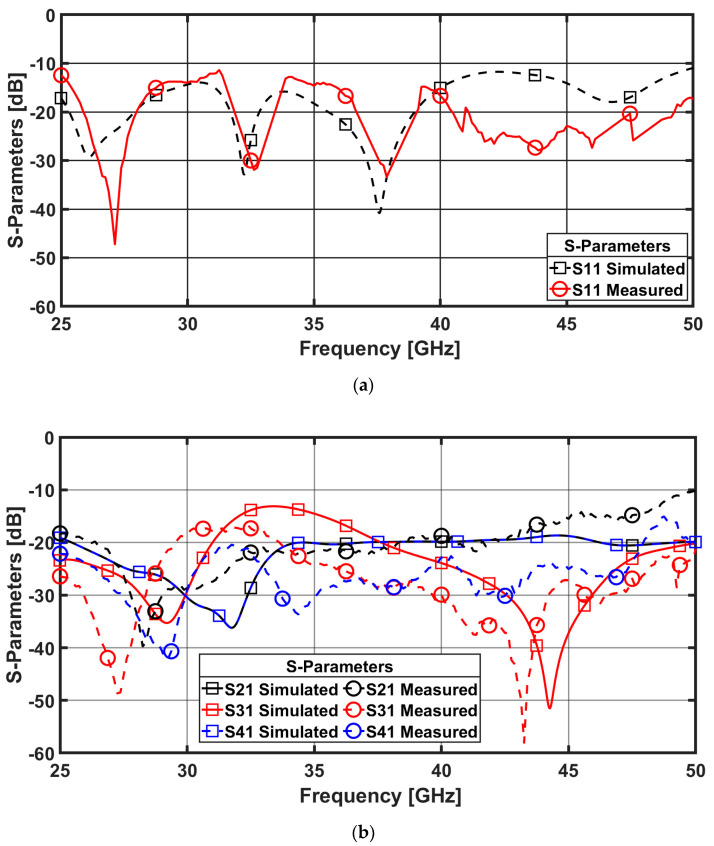
S-parameters coefficient (**a**) Reflection coefficient (**b**) Transmission coefficient.

The antenna is fabricated as shown in Figure 3 and measured using ROHDE & SCHWARZ ZVA67 vector Network Analyzer. A demonstration of the S-parameters coefficients values can be seen in Figure 6. It is clear that the reflection coefficient level is below −10 dB over the whole band from 25 to 50 GHz, as shown in Figure 6a. The difference between measurement and simulated results is noticeable in the higher frequency band due to the connectors in the millimetric band and in the other environments. The measurement setup is not in a completely shielded room. On the other hand, the coupling parameters are below −20 dB on average. It reaches −40 dB to −50 dB in individual sub-bands, as shown in Figure 6b.

### 3.1. Envelope Correlation Coefficient (ECC)

**Figure 7:**
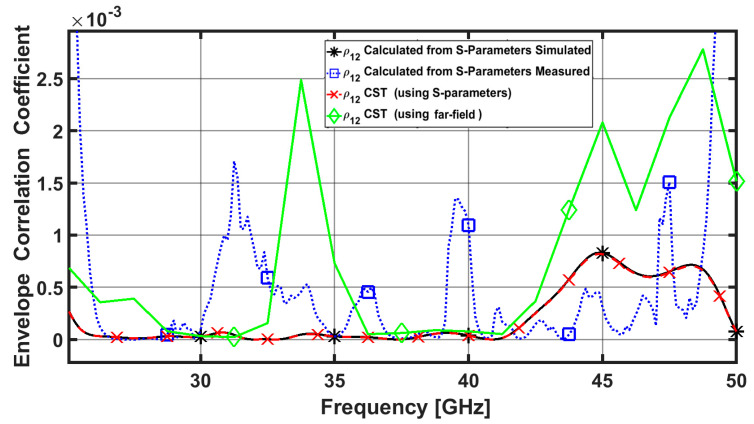
ECC of the proposed four-element MIMO antenna between port 1 and 2.

The level of correlation between various antenna components in a MIMO configuration is measured by ECC. Lower ECC results in less interdependence between the components and, as a result, better MIMO diversity performance. Equations (6) and (7), respectively, show how to compute the ECC from both the far field radiation pattern and the scattering parameters [28,35].

(6) $$\;\;\;\;\;\;\;\;\;\;\;ECC=\rho_{e}=\left|\rho_{ij}\right|=\frac{{\left|\int^{\;}\int^{\;}4\pi \left[F_{i}\left(\theta ,\phi \right)\right]•\left[F_{j}\left(\theta ,\phi \right)\right]d\Omega \right|}^{2}}{\int^{\;}\int^{\;}4\pi {\left|F_{i}\left(\theta ,\phi \right)\right|}^{2}d\Omega \int^{\;}\int^{\;}4\pi {\left|F_{j}\left(\theta ,\phi \right)\right|}^{2}d\Omega }$$

where i,j=1, 2, 3, 4, $\rho_{ij}$ is the ECC between $i^{th}$ and $j^{th}$ antenna elements, while Fi (θ, φ) is the 3D radiation pattern field with excitation at port ‘‘i’’ and ‘‘•’’ denotes the Hermitian product and ‘‘Ω’’ is the solid angle.

(7) $$ECC=\rho_{e}=\left|\rho_{ij}\right|=\frac{{\left|S_{ii}^{*}S_{ij}+S_{ji}^{*}S_{jj}\right|}^{2}}{\left(1-\left({\left|S_{ii}\right|}^{2}+{\left|S_{ji}\right|}^{2}\right)\right)\left(1-\left({\left|S_{jj}\right|}^{2}+{\left|S_{ij}\right|}^{2}\right)\right)}$$

where ‘‘∗’’ is the complex conjugate of the S-parameter. When assessing any lossy antenna, it is crucial to note that obtaining the ECC values using S-parameters is inaccurate and significantly underestimates its values. Therefore, because they are more accurate, the results acquired from far-field radiation patterns are more useful [36]. However, this process is complex and requires advanced calculations. If the ECC is lower than 0.5, then it is within the acceptable limit and the MIMO diversity is considered good as shown in Figure 7. Which indicates that no correlation between elements.

### 3.2. Diversity Gain (DG)

DG value is obtained from ECC, and it can be easily calculated using the following equation:

(8) $$DG=10*\sqrt{1-\rho_{ECC}}$$

**Figure 8:**
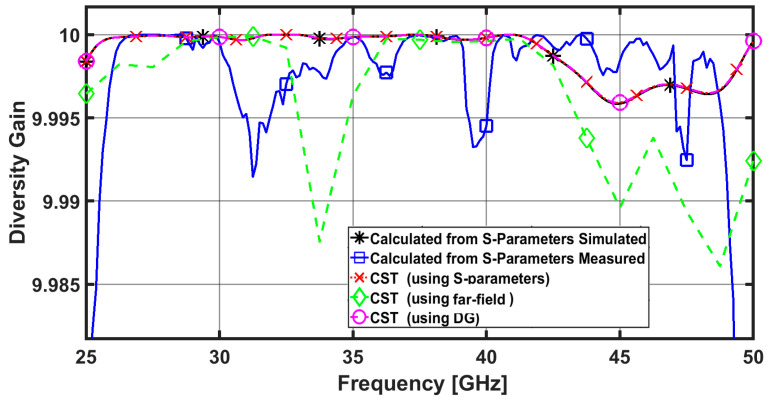
DG between ports 1 and 2 for the proposed four-element MIMO antenna.

It is clear that ECC and DG are inversely proportional to one another. A high DG value therefore indicates superior performance. The DG may be determined using both the far field radiation pattern and the scattering parameters, much like the ECC can [27]. Better isolation between the patch elements of the MIMO antenna is correlated with higher diversity gain values. More than 9.95 dB should be present in the DG. The estimated calculated values of the DG are higher than 9.99 dB across the antenna’s operating band, as shown in Figure 8. Which indicates the good diversity performance of the proposed MIMO antenna system.

### 3.3. Channel Capacity Loss (CCL)

The CCL parameter, which specifies the highest achievable limit of the information transmission rate, can be used to describe the channel capacity of MIMO systems. It calculates the ideal information transfer rate, in other words. An Equations from [37] can be used to derive the CCL parameter.

(9) $$C\left(Loss\right)=-\log_{2}\det(\psi^{R})$$

where $\psi^{R}$ indicates the correlation matrix at the receiving antenna.

(10) $$\psi^{R}=\left[\begin{array}{cc} \rho 11 & \rho 12\\ \rho 21 & \rho 22 \end{array}\right]$$

(11) $$\rho_{ii}=1-\left({\left|S_{ii}\right|}^{2}+{\left|S_{ij}\right|}^{2}\right),for\;\;i,j=1or2$$

(12) $$\rho_{ij}=-\left(S_{ii}{}^{*}S_{ij}+S_{ji}{}^{*}S_{ij}\right),for\;\;i,j=1or2$$

**Figure 9:**
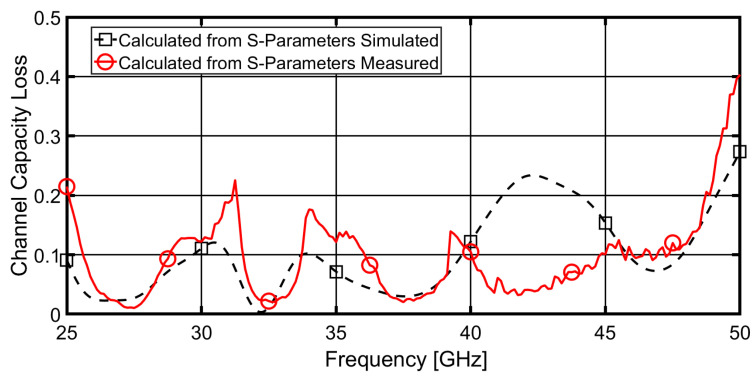
CCL of the proposed four-element MIMO antenna between port 1 and 2.

Figure 9 depicts the calculated and measured CCL. The CCL value is clearly less than 0.3 bps/Hz across the entire band. So, using the available CCL, it is possible to confirm that the proposed antenna provides a higher transmission data rate in any scattering environment.

### 3.4. Multiplexing Efficiency (ME)

**Figure 10:**
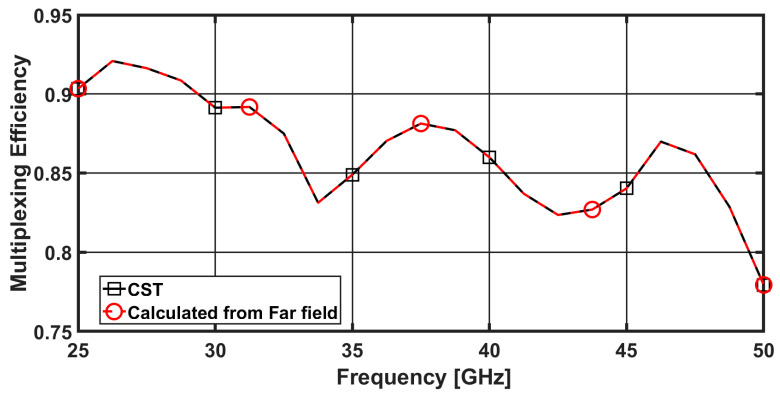
ME of the proposed four-element MIMO antenna between port 1 and 2.

ME is defined as the ratio between the power of the real antenna and that of the ideal one. The maximum ME has been calculated as [38]:

(13) $$\eta_{max}=\sqrt{\eta_{i}\eta_{j}\left(1-{\left|\rho_{eij}\right|}^{2}\right)}$$

where $\eta_{i}\;and\;\eta_{j}$ is the total efficiency of the $i^{th}$ and $j^{th}$ antenna elements and $\rho_{eij}$ is the magnitude of complex correlation coefficients between $i^{th}$ and $j^{th}$ antenna elements. The simulated and the measured ME are around −1 dB within the band from 25 GHz to 50 GHz, as illustrated in Figure 10. Which indicate that the MIMO antenna system are considered as ideal from 80% to 92% all over the band.

### 3.5. Total Active Reflection Coefficient (TARC)

The ratio between the square roots of the total power reflected and the total power incident, is known as the total active reflection coefficient (TARC). It may be estimated using the S-parameters and depicts the random signal combinations and mutual couplings between the ports. TARC is the sole MIMO parameter that takes into account the unpredictable phases of incoming signals, which can have a significant impact on MIMO array behavior in certain circumstances. TARC of 4-port MIMO antenna can be calculated as [39,40]:

(14) $$TARC=N^{-0.5}*\sqrt{\overset{N}{\underset{i=1}{\sum }}{\left|\overset{N}{\underset{k=1}{\sum }}S_{ik}e^{j\theta_{k-1}\;}\right|}^{2}}$$

where $S_{ik}$ is the scattering parameter between the $i^{th}$ and $k^{th}$ ports, and $\theta_{k-1}$ is the excitation phase difference between the $i^{th}$ and $k^{th}$ ports.

**Figure 11:**
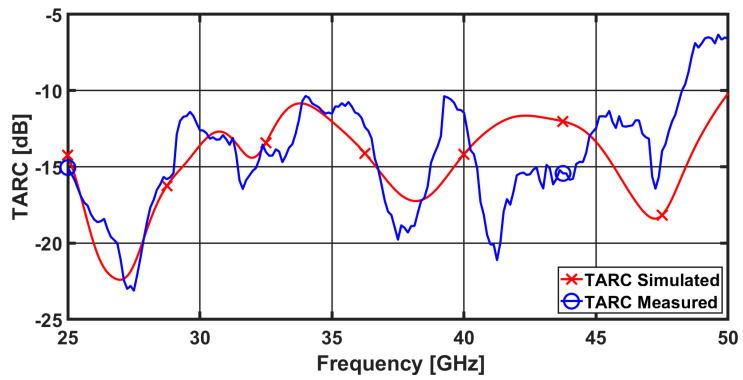
TARC simulated and measured results of the proposed four-element MIMO antenna.

The value of TARC for a MIMO communication system shouldn’t exceed 0 dB. Figure 11 displays the measured and simulated values of TARC. The value of the proposed antenna’s simulated and measured TARC is clearly under 10 dB for nearly the whole frequency range between 25 and 50 GHz where it is near to the reflection coefficients (the reference band), as can be seen from the Figure. The fabrication tolerance, test connectors, and connecting cables, as well as the surrounding test environment may all contribute to a slight difference between the measured and simulation findings.

## 4. Performance Comparison

In Table 1, a comparison between the proposed MIMO antenna system and the most recent MIMO work working in the 25–50 GHz region is made. The analysis takes several MIMO antenna characteristics into account. The proposed antenna with the same number of ports is more compact than [41,42,43]. In terms of realized gain, radiation efficiency, and isolation between antenna elements, it delivers superior MIMO performance than other small designs in [44,45,46]. Additionally, it has a high fractional bandwidth and a decreased mutual coupling. Its size is not the smallest, but compared to other literature work, it exhibits a considerable improvement in high isolation and superior MIMO diversity performance. This makes it possible to use the suggested antenna for 5G mm-wave applications. It’s also crucial to note that the proposed design is straightforward and has symmetric geometry, making it simple to include into larger MIMO antennas with more radiating elements.

## 5. Conclusions

The proposed MIMO antenna system has been designed to provide high isolation and UWB capability, with a perfect alignment orientation between the four elements and etching the ground using DGS. The proposed antenna system has achieved excellent performance parameters, such as low mutual coupling of less than −10 dB, low ECC of less than 0.005, low CCL of 0.25 bits/s/Hz, high DG of 9.999 dBi and low TARC of less than -10 dB, which shows a superior diversity performance. In addition, the wide operational bandwidth of 25 GHz, from 25 to 50 GHz, enables the antenna to be used in multiple applications. The compact design and low-cost design features make it suitable for mm-wave 5G applications and can be easily integrated into telecommunication devices. Furthermore, the suggested MIMO antenna system offers great performance and portability, making it an ideal choice for a wide range of 5G mm-wave applications.

## Figures and Tables

**Table 1:** Comparisons between the proposed MIMO antenna system and the published literature operating in 25–50 GHz band.

Reference	Physical Dimension (mm^2^), Number of Ports	Substrate $\varepsilon_{r}$ Thickness	Frequency, Band (B.W), Fractional B.W (GHz%)	MIMO Parameters					Mutual Coupling Reduction Technique
				ECC	DG	CCL	ME	TARC	
[41]	80 × 80 4 ports	Roger 5880, $\varepsilon_{r}=2.2$ 0.508	23–40, 17, 53%	<0.0014	NA	NA	NA	NA	DGS
[42]	23.75 × 42.5 4 Ports	Roger 5880, $\varepsilon_{r}=2.2$ 0.508	24–28, 4, 14.2%	<0.002	9.9	<0.001	N/A	N/A	DGS
[43]	45 × 45 4 Ports	FR-4 $\varepsilon_{r}=4.2$, 1.6	4.98–5.98, 1, 16.7%	<0.0022	9.95	<0.4	N/A	CALC.	Floating Parasitic Element
[44]	30 × 30 4 ports	Roger 5880, $\varepsilon_{r}=2.2$ 1.575	25–31, 6, 21.1%	<0.0005	NA	0.15	NA	NA	DGS
[45]	17 × 17 4 Ports	FR-4 $\varepsilon_{r}=4.2$, 0.8	3.4–3.6, 0.2, 5.5%	0.2	NA	57	CALC.	NA	Orthogonal Polarization
[46]	5 × 4 Single	Roger 5880, $\varepsilon_{r}=2.2$ 1.575	5.5–9.5, 4, 40%	N/A	N/A	N/A	N/A	N/A	N/A
*Proposed* *Antenna*	33 × 33 4 Ports	RO4003C, $\varepsilon_{r}=3.55$ 0.203	25–50, 25, 66%	<0.005	≈10	<0.25	>0.85	<−10	Orientation and DGS

## Data Availability

Not all data is applicable. However, the MATLAB portion of the data is useful and can be shared. To access this data, please reach out to the first author via email.
